# Internal fixation of acetabular fractures in an older population using the lateral-rectus approach: short-term outcomes of a retrospective study

**DOI:** 10.1186/s13018-018-1039-z

**Published:** 2019-01-04

**Authors:** Jiahui Chen, Han Liu, Canbin Wang, Xuezhi Lin, Cheng Gu, Shicai Fan

**Affiliations:** 1 Department of Orthopaedic, The Third Affiliated Hospital of Southern Medical University, Guangzhou, 510630 Guangdong Province China; 2 Department of Orthopaedic, Zhuhai People’s Hospital, Zhuhai, 519000 Guangdong Province China; 3 Department of Orthopaedic, Huadu District People’s Hospital of Guangzhou, Guangzhou, 510800 Guangdong Province China

**Keywords:** Lateral-rectus approach, Acetabular fracture, Elderly, Internal fixation, Three-dimensional printing

## Abstract

**Purpose:**

This study aims to examine the clinical efficacy and surgical techniques of the lateral-rectus approach for treatment of acetabular factures in elderly patients.

**Methods:**

After appropriate exclusion, 65 elderly patients with an acetabular fracture who was treated through the lateral-rectus approach from January 2011 and October 2016 were selected retrospectively. By analyzing the medical records retrospectively, the patients’ characteristics, fracture type, mechanism of injury, comorbid conditions, ASA class, operative time, intra-operative blood loss, and post-operative complications were assessed. Clinical examination radiographs have been taken, align with the Matta evaluation system. Functional outcomes were evaluated using surveys including SF-36, Harris hip score, and modified Merle D’Aubigne-Postel.

**Results:**

All 65 patients had undergone the single lateral-rectus approach successfully. Surgery duration was 101.23 min on average (45–210), and intra-operative bleeding was 798.46 ml on average (250–1800). According to the Matta radiological evaluation, the quality of reduction evaluated 1 week after surgery was rated as “anatomical” in 41 (63.1%) cases, “imperfect” in 12 (18.5%) cases, and “poor” in 12 (18.5%) cases. The modified Merle D’Aubigne-Postel score performed 18 months after surgery was categorized as excellent in 40 (61.5%) cases, good in 10 (15.4%) cases, and fair in 15 (23.1%) cases. The mean Harris Hip score was similar as present researches, being 87.18. The mean SF-36 score was 69.12 which was considered as normal for the group age 60 and older. Several complications were found, including screw loosening in 10 cases, fat liquefaction of incision in 2 cases, deep vein thrombosis in 2 cases, and temporary weakness of hip adductors in 5 cases. None of the patients had heterotopic ossification.

**Conclusions:**

The lateral-rectus approach is a valuable alternative to the ilioinguinal and modified Stoppa approach, being the treatment of acetabular fractures in elderly patients.

## Background

Acetabular fracture is an intra-articular fracture existing in the most important weight-bearing joints of humankind, and as a result, open reduction and rigid internal fixation of the displaced fracture fragments in time will lead to a better outcome than conservative treatment proposed by Judt et al. in the 1960s [1]. However, the acetabular fractures may be the most challenging fractures for the orthopedic trauma surgeons because of the special region of anatomy, irregular anatomy morphology, and a variety of fracture types of acetabulum [2, 3]. Moreover, there is an increasing prevalence of osteoporosis in the senior population, which has contributed to the fact that older patients become the fastest-growing group suffers from acetabular fractures. The difficulty and the risk of surgery are increased by their general condition, bone quality, and their restricted physiological reserves [2, 4–7].

In order to reduce postoperative pain, post-traumatic arthritis, failure of implants, to maximize retain original function and to remove complications, anatomical reconstruction of the original joint surface and concentric alignment of hip joint should be obtained. Accurate anatomic reduction and firm fixation can be obtained easily through a suitable approach providing an adequate operative field for the surgeons [2]. According to current studies, diverse traditional surgical approaches are used for surgery treatment of the acetabular fractures and they are categorized into anterior (the ilioinguinal approach, the modified Stoppa approach, the extended ilioinguinal approach, the Pararectus approach), posterior (Kocher-Langenbeck approach), and combined approach.

It is crucial to approach this treatment in an effective way. Mayo [8] proposed that there are five major considerations should be taken into account when the surgical approach is decided. The fracture pattern, age, and functional status are two of them. Both columns and anterior column fractures are the most common fracture pattern in the elderly patients [6]. Because the ilioinguinal approach can provide a wide view of the anterior column and the inner surface of the posterior column, it is the “gold standard” for the treatment of acetabular fractures in elderly patients. However, the ilioinguinal approach might be suboptimal because of the longer operating time, increased blood loss, and the high rate of soft tissue complications. The lateral-rectus approach, a novel anterior approach which provides adequate exposure of the anterior columns, the quadrilateral plate and the inner surface of posterior column with the less injury, is used for the complicated acetabular fractures in adults [9, 10]. And it may be a better choice for the elderly patients suffering acetabular fractures.

Consequently, from January 2011 to October 2016, 65 patients aged 60 years and older suffering acetabular fractures were surgically treated with the lateral-rectus approach. The aims of this study are to present surgical techniques of the reduction and fixation of fracture fragments via the lateral-rectus approach and to evaluate the clinical efficacy.

## Materials and methods

### Study participants

#### Inclusion criteria

1. Aged 60 and older patients
2. Suffering from acetabular fractures with anterior column
3. The time between injury to surgery is less than 2 weeks
4. Surgically treated with the single lateral-rectus approach

#### Exclusion criteria

1. The fractures without significant dislocation
2. The Ipsilateral abdomen have suffered surgery
3. Associated with posterior wall fracture needed to be fixed via posterior approach
4. Treated through an anterior-posterior combined approach or a conservative treatment

### Perioperative management

The related routine and preoperative examinations were performed immediately after the patients’ confirmation. Skeletal traction of distal femur was used on the affected side with 1/7–1/8 weight of the patient, and the Rivaroxaban (10 mg, qd) was given to prevent the deep vein thrombosis. In addition, the pre-existing comorbidities were systemically treated through cooperating with the related physicians. To ensure the patients did not suffer from the deep vein thrombosis, the Doppler vascular examination was performed again 1 day before the operation. Withholding oral ingestion of food and fluids for 8 h and cleansing enema was performed the night before the operation day.

**Fig. 1:**
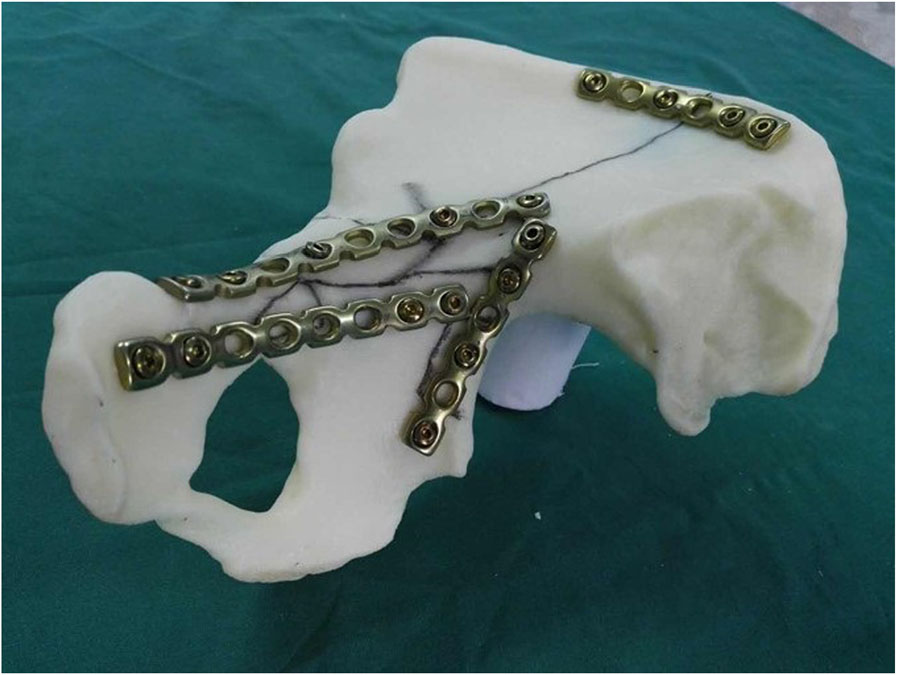
The key fracture line was marked on the mirror model, and the positions and the length of the plates were confirmed based on the fracture patterns

Standard pelvic CT scan (Toshiba 64 lines, 1 mm) were performed in all the patients and then inputted the original data (Dicom standard) into Mimics software (Materialise, 15.0). After three-dimensional reconstruction through Mimics software, 1:1 3D (three-dimensional) pelvic models were manufactured by 3D printer (Stratasys Dimension 1200es), which can clearly and certainly indicate the fracture types, the positions of fractures, and the displacements from different angles. A mirror pelvic model of the uninjured side was also created by 3D printer. The key fracture lines were marked on the mirror model, and the positions and the length of the plates were confirmed based on the fracture patterns. The plates were then reshaped to ensure it accommodated the model bone surface. The positions, length, and direction of the screws were also identified and recorded (Fig. 1). Finally, the pre-shaped plates were used in the operations after being routinely sterilized.

A broad-spectrum antibiotic was given 30 min before the operation, if the operative time was more than 3 h or intra-operative blood loss more than 1000 ml, the antibiotic would be given again. An indwelling drainage tube was used after the operation and would be removed when the drainage volume became less than 50 ml daily. Liquid diet was allowed after the passage of gas by anus, and the laxative was given to prevent constipation if necessary. Three days after the surgery, active and passive activities of ipsilateral lower limb were allowed. Patients were permitted to partial-weight bearing in 4 to 6 weeks, and full-weight bearing in 8 to 12 weeks.

### Surgical technique

**Fig. 2:**
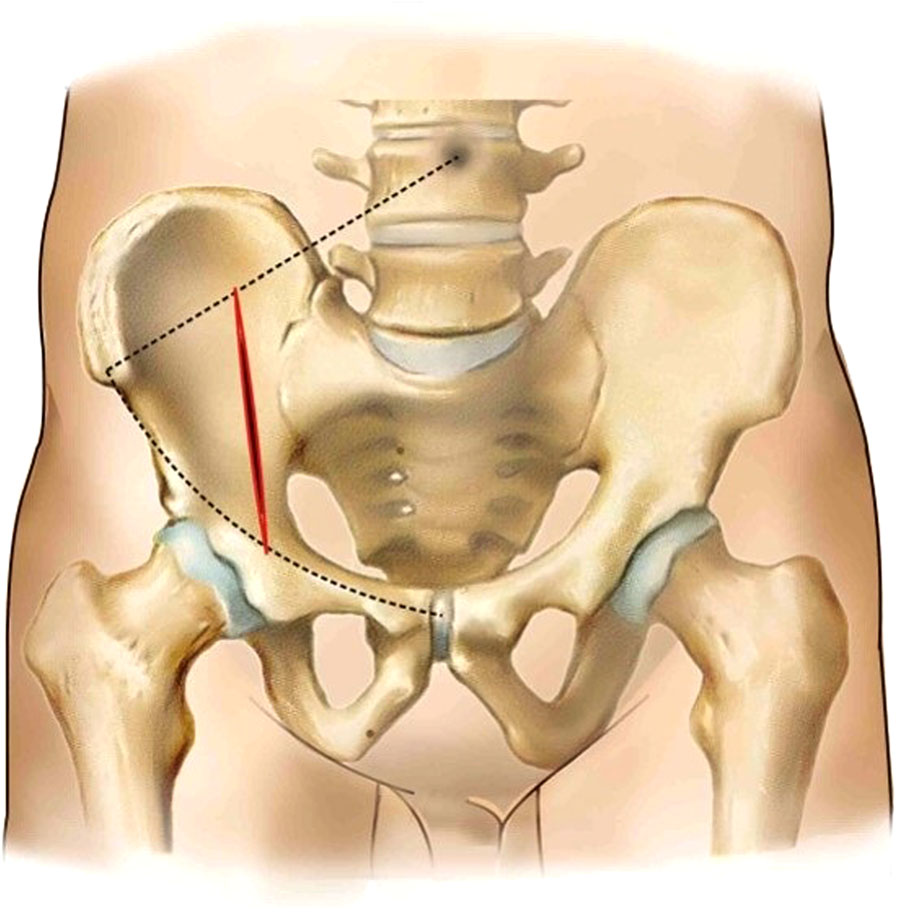
The incision of the lateral-rectus approach starts at the point located two thirds of the distance from the umbilicus to the anterior superior iliac spine and end at the midpoint of the inguinal ligament

**Fig. 3:**
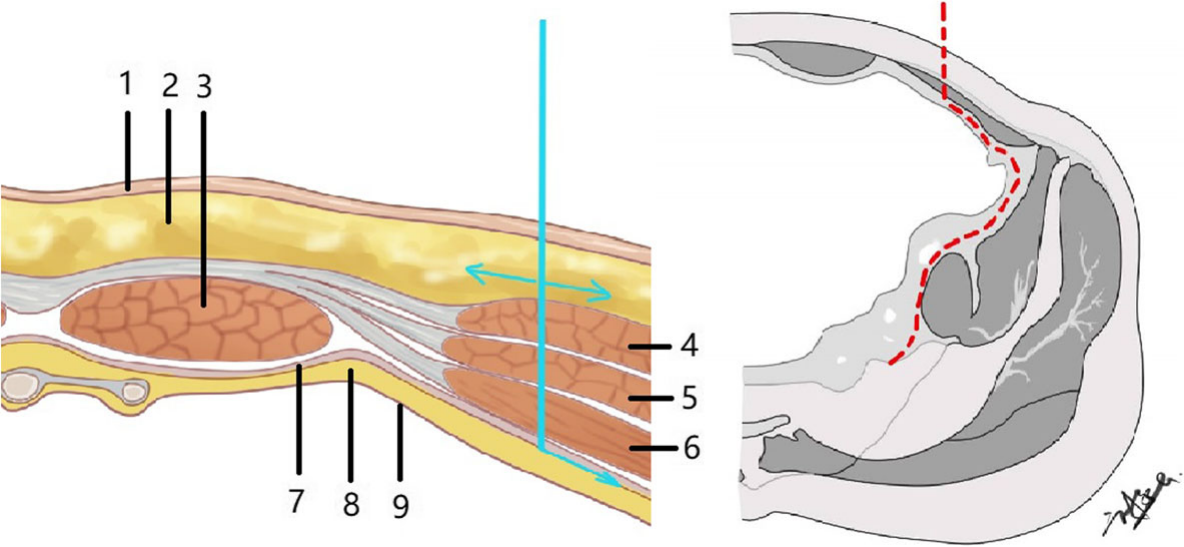
Diagram of the cross-sectional anatomy of the surgical approach with the direction of dissection: 1 skin, 2 subcutaneous tissue, 3 rectus abdominis, 4 obliquus externus abdominis, 5 obliquus internus abdominis, 6 transverse abdominis, 7 transversalis fascia, 8 extraperitoneal space, 9 peritoneum

Patients were placed in the supine position on a radiolucent operating table after tracheal intubation general anesthesia. The incision of the lateral-rectus approach starts at the point located two thirds of the distance from the umbilicus to the anterior superior iliac spine and end at the midpoint of the inguinal ligament. The incision is along the lateral border of the rectus abdominis muscle and about from 8 to 10 cm long (Fig. 2). The incision can be extended upward if there is not enough exposure of fractures. The extraperitoneal space is entered after subcutaneous dissection and oblique split of the obliquus externus abdominis, obliquus internus abdominis, transverse abdominis, and ends at the medial margin of the superficial inguinal ring (Fig. 3).

**Fig. 4:**
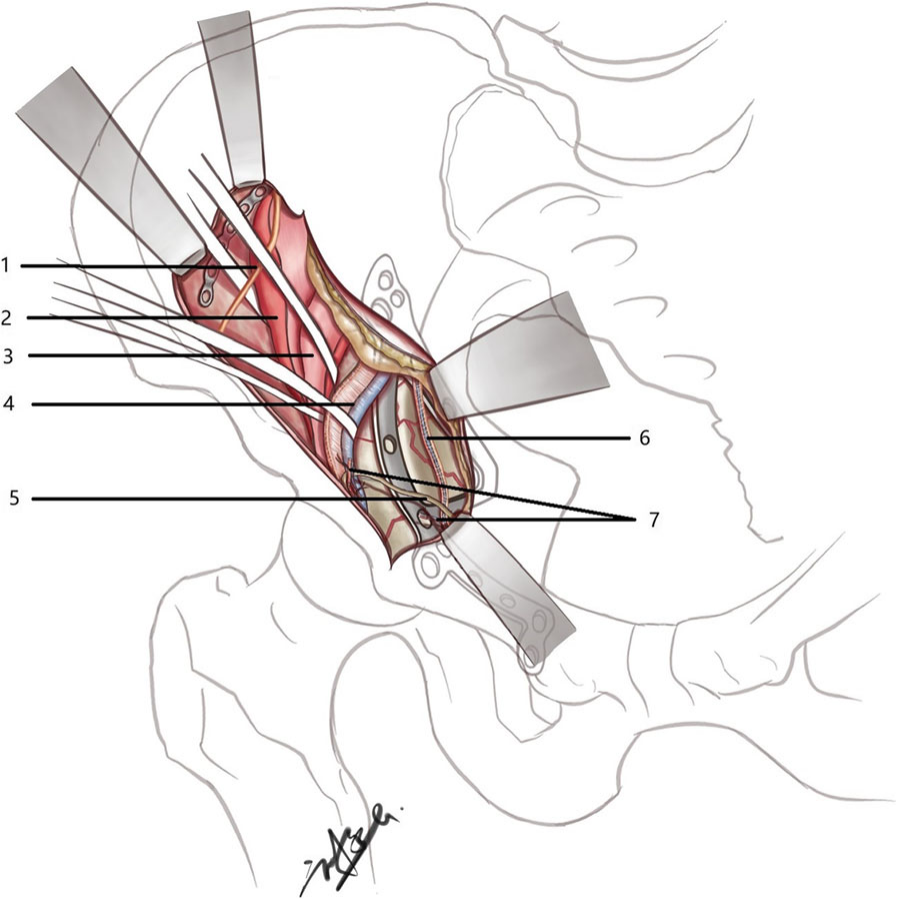
Diagram of the surgical exposure, showing 1 lateral femoral cutaneous nerve, 2 iliacus muscle, 3 psoas major muscle, 4 external iliac artery/vein, 5 vas deferens, 6 obturator nerve and vessels, and 7 anastomosis between obturator and inferior epigastric or external iliac vessels (corona mortis, ligated)

The first operative window is between the peritoneum and femoral vessels where the pubic branch, obturator foramen, corona mortis, quadrilateral plate, anterior column, and anterior wall can be exposed clearly. After ligaturing the corona mortis to prevent bleeding, we can reduce and fix the fracture of the anterior column, quadrilateral plate, and anterior wall in this operative window. Another window is between iliac vessels and iliopsoas muscle. This window could expose anterior column of acetabulum, quadrilateral plate, alae sacralis, small pelvic ring, and even ischial spine in deep position. It cannot only reduce and fix the fracture of small pelvic ring, displacement of the quadrilateral area, and fracture around sacroiliac joint conveniently, but also reduce the fracture of posterior column visibly from the inner surface of the posterior column. The traction for obturator nerve is inevitable when exposing quadrilateral plate and ischial spine because obturator nerve is close to the medial surface of the acetabulum. Therefore, to avoid damaging the nerve, the operation needs to be performed carefully (Fig. 4).

### Follow-up and evaluation criteria

Follow-up including radiographic and hip function assessment was completed at 6 weeks, 12 weeks, 6 months, and 18 months after surgery. Quality of reduction was estimated on the AP pelvis and Judet pelvis view by the Matta evaluation system [11]. Functional outcome was evaluated using surveys including SF-36 [12], Harris Hip score [13], and modified Postel Merle D’Aubigne [11, 14]. All the evaluations were assessed by two independent orthopedic surgeons not involved in the definitive care or surgery.

## Result

During January 2011 and October 2016, 65 cases of acetabular fractures were included in this study. There were 42 males and 23 females, with an average age of 67.14 years (60–88) old. The initial injury cause was traffic accident in 28 cases, fall from height (greater than standing) in 28 cases, and fall (from standing height) in 9 cases. According to the classification system described by Judt et al., there were 30 cases of anterior column with posterior hemitransverse fractures, 21 cases of both-column fractures, 8 cases of T-type fractures, and 6 cases of anterior column fractures. In addition, most of them had multiple injuries, including ipsilateral pelvic ring fractures in 17 cases, extremity fractures in 14 cases, spine fractures in 4 cases, pleural effusion in 4 cases, and craniocerebral injury in 4 cases. Most of them also suffered from pre-existing comorbidities, including hypertension in 13 cases, coronary heart disease in 6 cases, diabetes mellitus in 6 cases, and lung disease in 5 cases. The fitness of patients before surgery was assessed based on ASA class [15]. There were 5 patients in class 1, 23 patients in class 2, 35 patients in class 3, 2 patients in class 4, and no patients in class 5 or 6. The time to surgery was 7.32 days on average (5–14). Patients’ demographics and characteristics are shown in Table 1.

**Table 1:** Patients’ demographics, mechanisms of injury, fracture classifications, multiple injuries, pre-existing comorbidity, ASA class, and time to surgery in a series of 65 patients (mean ± standard deviation (range) or number (percentage))

Parameter	Value
Age (years)	67 ± 5 (60–88)
Male gender	42 (64.6)
Mechanism of injury	
Traffic accident	28 (43.1)
Fall from height (greater than standing)	28 (43.1)
Fall (from standing height)	9 (13.8)
Fracture classification	
Anterior column with posterior hemitransverse	30 (46.2)
Both-column	21 (32.3)
T-type	8 (12.3)
Anterior column	6 (9.2)
Multiple injuries	
Ipsilateral pelvic ring fracture	17 (26.2)
Extremity fracture	14 (21.5)
Spine fracture	4 (6.2)
Pleural effusion	4 (6.2)
Craniocerebral injury	4 (6.2)
Pre-existing comorbidity	
Hypertension	13 (20.0)
Coronary heart disease	6 (9.2)
Diabetes mellitus	6 (9.2)
Lung disease	5 (7.7)
ASA class	
1	5 (7.7)
2	23 (35.4)
3	35 (53.8)
4	2 (3.1)
Time to surgery (days)	7 ± 2 (5–14)

All 65 patients underwent the operation via the single lateral-rectus approach successfully with an average of 7.32 days (5–14) after they were injured. Surgery duration was 101.23 min on average (45–210). Intra-operative bleeding was 798.46 ml on average (250–1800). According to the X-ray performed 1 week after the surgery, the quality of reduction was rated as “anatomical” in 41 (63.1%) cases, “imperfect” in 12 (18.5%) cases, and “poor” in 12 (18.5%) cases. Screw loosening occurred in 3 cases 2 weeks after the operation and 7 cases 3 months after the operation. In these 10 cases, fracture dislocation occurred in only 2 cases. Fat liquefaction of incision occurred in 2 cases which were cured by dressing changes. Deep vein thrombosis of affected side occurred in 2 cases 2 weeks after the operation, the filters of inferior vena cava was placed in one case by the vascular surgeon, and another case was treated by anti-thrombotic therapy. Embolus detachment did not occur in both cases. Five patients were noted with temporary weakness of hip adductors postoperatively. The injury of the lateral femoral cutaneous nerve and obturator nerve did not occur in any case. At the final follow-up, all the patients gained bone union and none of them had heterotopic ossification.

The average modified Merle d’Aubigne-Postel score evaluated 18 months after the surgery was 16.54 (12–18), categorized as excellent in 40 (61.5%) cases, good in 10 (15.4%) cases, fair in 15 (23.1%) cases, and poor in no cases. The average Harris Hip score evaluated 18 months after the surgery was 87.18, categorized as excellent in 40 (61.5%) cases, good in 20 (30.8%) cases, fair in 4 (6.2%) cases, poor in 1 (1.5%) case, and failed in no cases. The average physical component summary and mental component summary of SF-36 evaluated 18 months after surgery were 68.82 and 69.42 respectively. Operative time, intra-operative blood loss, radiographic and hip function outcomes, and complications are shown in Table 2.

**Table 2:** Operative time, intra-operative blood loss, radiographic and hip function outcomes, and complications (mean ± standard deviation (range) or number(percentage))

Parameter	Value
Operative time (minutes)	101 ± 27 (45–210)
Intra-operative blood loss (ml)	798 ± 322 (250–1800)
Complication	
Deep vein thrombosis	2 (3.1)
Fat liquefaction of incision	2 (3.1)
Screw loosening	10 (15.4)
Temporary weakness of hip adductors	5 (7.7)
Radiological outcome (Matta)	
Anatomical (< 1 mm)	41 (63.1)
Imperfect (1-3 mm)	12 (18.5)
Poor (> 3 mm)	12 (18.5)
Merle D’Aubigne-Postel score	
Excellent (18)	40 (61.5)
Good (15–17)	10 (15.4)
Fair (13–15)	15 (23.1)
Harris hip score	
Excellent (90–100)	40 (61.5)
Good (80–89)	20 (30.8)
Fair (70–79)	4 (6.2)
Poor (60–69)	1 (1.5)
SF-36	
Physical component summary	68.82 (± 10.67)
Mental component summary	69.42 (± 10.83)

## Discussion

As we all know, open reduction and internal fixation of the displaced fracture fragments in time will lead to a better outcome than that of the conservative treatment [3, 5, 16]. But accurate reduction and rigidly internal fixation of acetabular fractures are difficult because of its complicated anatomical structure and deep location. Therefore, it is necessary to perform a thorough radiographic analysis to determine a preoperative plan including the surgical approach and the order of reduction.

### Surgical requirements and techniques

**Fig. 5:**
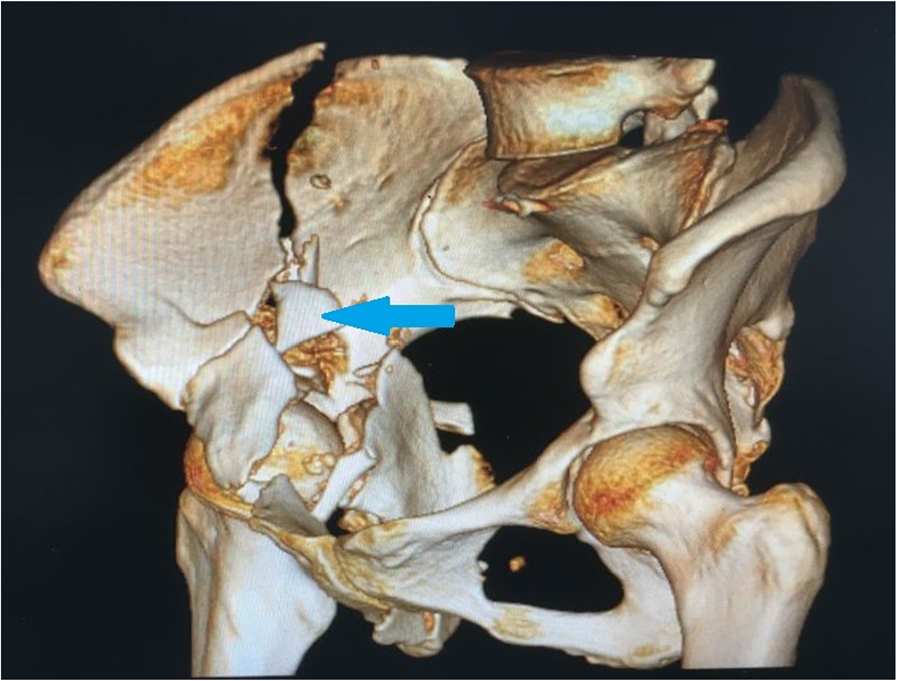
The key bone (blue arrows) in the sciatic notch above the acetabulum is what the author reduces first in this study

1. The surgical team should have rich experience to ensure the surgery is finished in a minimum time and perioperative complications are minimized.
2. Surgery approach should be selected, based on the fracture patterns and the surgeons’ proficiency of each surgical approach. If the surgery can be finished via a single approach, the combined approach should not be considered.
3. Due to the deep anatomical site and the rich muscle tissue, soft tissue was often embedded in the fracture ends. After exposing the fracture ends, we should thoroughly remove the soft tissue embedded in the fracture ends, and then reduce the bone fragments under the periosteum.
4. Fracture reduction order: acetabular fractures have varying degrees of comminution, which can cause complexity and difficulty in reducing the bone segments. The correct fractures order can help us to shorten the operation time and achieve the desired reduction effect. The key bone in the sciatic notch above the acetabulum (Fig. 5) is what the author reduces first in this study, and the quality of the bone improves. This can restore the anatomical signs and provide a fulcrum to reduce other bone segments.
5. Because of the poor bone quality in the elderly patients, merely using screws and plates to reduce the bone segments is not sufficient. Moreover, we should insert the screws precisely to avoid adjusting the screws repeatedly (usually has only one chance to insert screws in the elderly) and use locking plates to prevent screws from loosening.

### Therapeutic effect evaluation

By using the lateral-rectus approach in elderly patients with acetabular fractures, good functional and radiological results and a low complication rate can be achieved. Therefore, we believe that the lateral-rectus approach represents a viable alternative to the ilioinguinal approach in elderly patients.

All fractures were observed union in the final follow-up. The mean operative time, being 101.23 min, in our sample treatment via the lateral-rectus approach is significantly shorter than the operative time of procedures published previously. Ma et al. [17] identified operative times of 183 and 256 min in their study comparing the modified Stoppa approach with the ilioinguinal approach respectively. A study with Pararectus approach reports mean operative time of 200 min and mean blood loss of 1477ml [18]. The mean intra-operative blood loss of 798.46 ml in our study is similar to 776 ml of the modified Stoppa approach but less than 1107 ml of the ilioinguinal approach. A recently published system review by Daurka et al. [5] reported a mean operative time of 233 min and a mean blood loss of 891 ml in traditional approaches.

The anatomical reduction of 63.1% in our sample treatment via the lateral-rectus is better than what has been reported by Ma et al. and Daurka et al. The anatomical reduction rates are 53.3% of the modified Stoppa approach and 43.3% of ilioinguinal approach reported by Ma et al. 45.3% anatomical reduction rate of traditional approach is reported by Daurka et al. The excellent rate of modified Merle d’Aubigne-Postel score of 61.5% in our sample is better than the 43.3% of the modified Stoppa approach and 33.3% of the ilioinguinal approach reported by Ma et al. The mean modified Merle d’Aubigne-Postel score is 16.54 similar to the 16.1 reported by Daurka et al. This study’s mean Harris hip score of 87.18 is also comparable to Laflamme’s [19] mean Harris hip score of 86.2. The mean physical component summary and mental component summary of SF-36 are 68.82 and 69.42 respectively, which can be considered normal for the group age 60 and older in China [20].

### The advantages of the lateral-rectus approach

In general, better results are dependent on better radiographic results in the acetabular fractures [21]. The broad visual and tactile exposure to the fractures are crucial to the reduction quality. The ilioinguinal approach is the most common approach for the treatment of acetabular fractures in the elderly patients because of its advantage of providing a wide view of the anterior column and the inner surface of the posterior column. However, it is often associated with some soft tissue complications including hernias, lesions of the femoral vessels, thrombosis, hematoma, and impaired wound healing [17, 22]. The lateral-rectus approach also can expose the anterior column and the inner surface of the posterior column clearly with a low rate of complications. The incision and surgical procedure are both longitudinal and consistent with the direction of the external iliac vessels, so that we can avoid excessive traction of the blood vessels and lower the risk of postoperative deep vein thrombosis. The quadrilateral plate can be exposed clearly through this approach, so that the quadrilateral area can be reduced under direct vision and the bone-crushing caused by indirect reduction can be avoided. The sacroiliac joint also can be exposed clearly, so that the pre-sacral venous plexus hemorrhage can be treated.

The modify Stoppa approach was introduced as the method for approaching the anterior acetabular fractures by Hirvenaslo [23]. It provides the advantages of direct visualization of the entire pelvic brim from the pubic body to the anterior aspect of the sacral ala, direct visualization and access to the quadrilateral plate allowing for reduction and plating, and direct visualization and access to the posterior column from the greater sciatic notch to the ischial spine allowing for reduction and plating [24]. However, it cannot allow surgeons to reduce and fix the high anterior column and iliac fossa fractures [25]. By contrast, the lateral-rectus approach can expose the significantly large part of the inner surface of iliac fossa, so that both columns fractures associated with high iliac crest fractures can be treated via a single lateral-rectus approach.

**Fig. 6:**
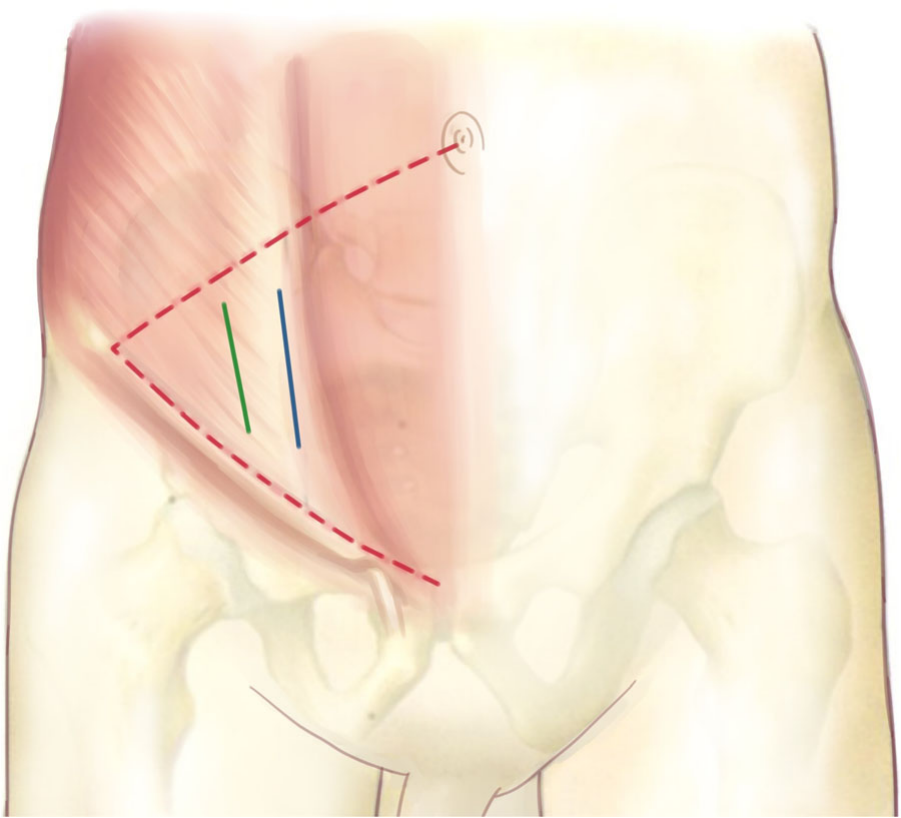
In order to enter extraperitoneal space, the rectus sheath is incised at the lateral border of rectus abdominis in using the Pararectus approach (blue line). Instead, obliquus externus abdominis, obliquus internus abdominis, and transverse abdominis are incised in using the lateral-rectus approach (green line)

Our lateral-rectus approach is similar to the Pararectus approach which reported by Keel [26]. There are still some differences between these two approaches. The lateral approach locates more lateral than the Pararectus approach (Fig. 6), so that it can expose the fractures of the iliac crest and high anterior column more clearly. It also provides more space to place a posterior column lag screw. Moreover, the lateral-rectus approach which incises muscles instead of rectus sheath may reduce the risk of hernia. But a large sample randomized controlled trial is needed to identify the differences.

### The limitations of the study

This study has some limitations including its retrospective design, lack of control group, few reported cases, and short follow-up time.

## Conclusion

The lateral-rectus approach, with good exposure of the anterior acetabulum and good clinical efficacy described in this study, is a viable alternative as it is a less invasive procedure for treating acetabular fractures in elderly patients. But clinical trials are necessary for the evaluation of mid- and long-term results to confirm its effectiveness and practicability.

## References

[1] Judt R, Judet J, Letournel E (1964). Fractures of the acetabulum: classification and surgical approaches for open reduction. Preliminary report. J Bone Joint Surg Am.

[2] Guerado E, Cano JR, Cruz E (2012). Fractures of the acetabulum in elderly patients: an update. Injury.

[3] Mayo KA. Open reduction and internal fixation of fractures of the acetabulum. Results in 163 fractures. Clin Orthop Relat Res. 1994;305(305):31–7. 8050243

[4] Clement ND, Court-Brown CM. Elderly pelvic fractures: the incidence is increasing and patient demographics can be used to predict the outcome. Eur J Orthop Surg Traumatol. 2014;24(8):1431–7. 10.1007/s00590-014-1439-7 24664452

[5] Daurka JS, Pastides PS, Lewis A, et al. Acetabular fractures in patients aged > 55 years: a systematic review of the literature. Bone Joint J. 2014;96-B(2):157–63. 10.1302/0301-620X.96B2.32979 24493178

[6] Ferguson TA, Patel R, Bhandari M, Matta JM. Fractures of the acetabulum in patients aged 60 years and older: an epidemiological and radiological study. J Bone Joint Surg Br. 2010;92(2):250–7. 10.1302/0301-620X.92B2.22488 20130318

[7] Yoshihara H, Yoneoka D. Trends in the incidence and in-hospital outcomes of elective major orthopaedic surgery in patients eighty years of age and older in the United States from 2000 to 2009. J Bone Joint Surg Am. 2014;96(14):1185–91. 10.2106/JBJS.M.01126 25031373

[8] Mayo KA. Surgical approaches to the acetabulum. Tech Orthop. 1990;4(4):24–35.

[9] Xiao Z, Xiao-dong Y, Guang X, et al. Surgical treatment of complex acetabular fractures through the lateral-rectus approach with the pelvic reconstructive plate and antegrade posterior-column lag screw. J Trauma Surg. 2015;17(2):123–6.

[10] Xiaodong Y, Guang X, Shicai F, et al. Operative treatment of acetabular both-column fracture through single lateral-rectus approach. 2015;35(4):335–340.

[11] Matta JM. Fractures of the acetabulum: accuracy of reduction and clinical results in patients managed operatively within three weeks after the injury. J Bone Joint Surg Am. 1996;78(11):1632–45. 8934477

[12] Ware JJ, Sherbourne CD. The MOS 36-item short-form health survey (SF-36). I. Conceptual framework and item selection. Med Care. 1992;30(6):473–83. 1593914

[13] Harris WH. Traumatic arthritis of the hip after dislocation and acetabular fractures: treatment by mold arthroplasty. An end-result study using a new method of result evaluation. J Bone Joint Surg Am. 1969;51(4):737–55. 5783851

[14] D'Aubigne RM, Postel M. Functional results of hip arthroplasty with acrylic prosthesis. J Bone Joint Surg Am. 1954;36-A(3):451–75. 13163078

[15] Anoymous. New classification of physical status. Anesthesiology. 1963;24:111.

[16] Carroll EA, Huber FG, Goldman AT, et al. Treatment of acetabular fractures in an older population. J Orthop Trauma. 2010;24(10):637–44. 10.1097/BOT.0b013e3181ceb685 20871252

[17] Ma K, Luan F, Wang X, et al. Randomized, controlled trial of the modified Stoppa versus the ilioinguinal approach for acetabular fractures. Orthopedics. 2013;36(10):e1307–15. 10.3928/01477447-20130920-25 24093709

[18] Keel MJ, Tomagra S, Bonel HM, et al. Clinical results of acetabular fracture management with the Pararectus approach. Injury. 2014;45(12):1900–7. 10.1016/j.injury.2014.10.040 25457342

[19] Laflamme GY, Hebert-Davies J, Rouleau D, et al. Internal fixation of osteopenic acetabular fractures involving the quadrilateral plate. Injury. 2011;42(10):1130–4. 10.1016/j.injury.2010.11.060 21156315

[20] Li N, Liu C, Li J, Ren X. The norms of SF-36 scale scores in urban and rural residents of Sichuan province. Hua Xi Yi Ke Da Xue Xue Bao. 2001;32(1):43–7. 12733352

[21] Jr BJ, Goldfarb C, Ricci W, et al. Functional outcome after isolated acetabular fractures. J Orthop Trauma. 2002;16(2):73–81. 10.1097/00005131-200202000-00001 11818800

[22] Ochs BG, Marintschev I, Hoyer H, et al. Changes in the treatment of acetabular fractures over 15 years: analysis of 1266 cases treated by the German Pelvic Multicentre Study Group (DAO/DGU). Injury. 2012;41(8):839–51. 10.1016/j.injury.2010.04.010 20451195

[23] Hirvensalo E, Lindahl J, Böstman O. A new approach to the internal fixation of unstable pelvic fractures. Clin Orthop Relat Res. 1993;297:28–32. 8242945

[24] Yong KH, Yang DS, Kyu PC, Sik CW. Modified Stoppa approach for surgical treatment of acetabular fracture. Clin Orthop Surg. 2015;7(1):29–38. 10.4055/cios.2015.7.1.29 PMC4329530 25729516

[25] Rocca G, Spina M, Mazzi M. Anterior Combined Endopelvic (ACE) approach for the treatment of acetabular and pelvic ring fractures: a new proposal. Injury. 2014;45(6):S9–15. 10.1016/j.injury.2014.10.016 25457312

[26] Keel MJ, Ecker TM, Cullmann JL, et al. The Pararectus approach for anterior intrapelvic management of acetabular fractures: an anatomical study and clinical evaluation. J Bone Joint Surg. 2012;94(3):405–11. 10.1302/0301-620X.94B3.27801 22371551

